# Value of anal swabs for SARS-COV-2 detection: a literature review

**DOI:** 10.7150/ijms.59382

**Published:** 2021-04-16

**Authors:** Yuliang Wang, Xiaobo Chen, Feng Wang, Jie Geng, Bingxu Liu, Feng Han

**Affiliations:** 1 Department of Clinical Laboratory Medicine, the Second Hospital of Tianjin Medical University, Tianjin Institute of Urology, Tianjin, China.; 2 Unicell Life Science Development Co., Ltd, Tianjin, China.; 3 Department of Genetics, School of Basic Medical Sciences, Tianjin Medical University, Tianjin, China.; 4 Department of rheumatology and immunology, Tianjin medical university general hospital, Tianjin, China.

**Keywords:** coronavirus disease 2019, severe acute respiratory syndrome coronavirus 2, anal swab, nucleic acid, screening, asymptomatic infections, close contact.

## Abstract

Facing the unprecedented global public health crisis caused by coronavirus disease 2019 (COVID-19), nucleic acid tests for severe acute respiratory syndrome coronavirus 2 (SARS-CoV-2) are the gold standard for diagnosing COVID-19. The asymptomatic carriers were not suspected of playing a significant role in the ongoing pandemic, and universal nucleic acid screening in close contacts of confirmed cases and asymptomatic carriers has been carried out in many medium- and high-risk areas for the spread of the virus. Recently, anal swabs for key population screening have been shown to not only reduce missed diagnoses but also facilitate the traceability of infectious sources. As a specimen for the detection of viruses, the goal of this paper is to briefly review the transmission route of SARS-CoV-2 and the necessity of using anal swabs for SARS-CoV-2 screening to minimize transmission and a threat to other people with COVID-19.

## Introduction

Coronavirus disease 2019 (COVID-19) can spread globally at a very rapid rate due to globalization and increased international travel and trade. With our increasing knowledge of this disease, the COVID-19 pandemic has the potential to change the paradigm of health care delivery, social/lifestyle habits and rhythm, as well as mood changes 1-3. As global cases of the novel coronavirus SARS-CoV-2 result in more than 100 million cases and 2.3 million deaths, it is accepted that far-reaching restrictions and waves of infection will be recurrent in the foreseeable future 4. According to statistics, the mean incubation period of COVID-19 was estimated to be 5.2 days, and 95% of the distribution of the incubation period was 12.5 days, indicating that the incubation period varied greatly among patients, which depended upon the patient's immunological conditions 5. With the stable and controllable state of the COVID-19 epidemic in China, rapid and effective virus nucleic acid screening has been essential for the timely diagnosis of confirmed and suspected patients.

Notably, some asymptomatic carriers or patients with mild existing symptoms continued to display active viral infection up to several days after clearance of SARS-CoV-2 from respiratory samples 6. Several studies have shown that a notable proportion of patients with COVID-19 develop gastrointestinal symptoms, and nearly half of patients confirmed to have COVID-19 have shown detectable SARS-CoV-2 RNA in their fecal or anal swab samples. Furthermore, studies have also found that the duration of nucleic acid-positive stool or anal swabs of some infected people is longer than the duration of the upper respiratory tract 7, 8. Therefore, increasing the detection of anal swab nucleic acids can increase the detection rate of asymptomatic carriers and reduce missed diagnoses. At present, nasopharyngeal swabs and oropharyngeal swabs have been widely used in mass SARS-CoV-2 screening. As a specimen for the detection of viruses, the goal of this paper is to briefly review the transmission route of SARS-CoV-2 and the necessity of using anal swabs for SARS-CoV-2 screening to minimize transmission and a threat to other people with COVID-19.

## Transmission route of SARS-CoV-2

**Figure 1:**
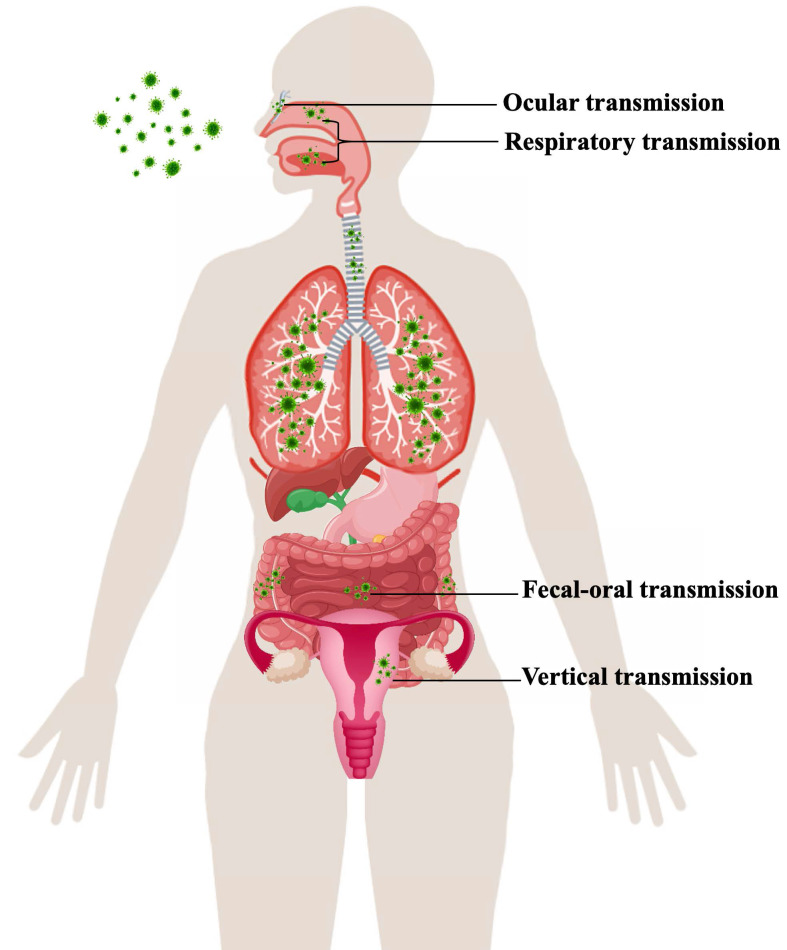
Transmission route of SARS-CoV-2.

The reason why COVID-19 was characterized as a pandemic by the World Health Organization (WHO) on March 11, 2020, is that SARS-CoV-2 has exhibited strong people-to-people transmissibility and has spread rapidly across countries 9. Transmission routes for SARS-CoV-2 are still not completely elucidated. Chinese and World Health Organization experts completed their work in Wuhan, which is part of global scientific research on the origin of SARS-CoV-2, according to a joint WHO-China press conference on Feb 9, 2021. The joint study said that although an intermediary host species is “the most likely” pathway, direct transmission or introduction through cold-chain food is also likely. However, a laboratory incident is “extremely unlikely” as the cause of COVID-19. An understanding of the virus's various modes of transmission and infection of person-to-person is required for its effective containment (Figure 1).

### Respiratory transmission

SARS-CoV-2 is predominantly characterized as a respiratory tract infection. The virus commonly presents from nonspecific lower respiratory tract infection symptoms, such as fever, cough, and shortness of breath, to severe pneumonia, which might be aggravated to acute respiratory distress syndrome (ARDS) or multiorgan dysfunction (MOD) 10. Environmental contamination through airflow may perpetuate viral transmission via infectious respiratory droplet nuclei formed while coughing, sneezing, singing, breathing, and speaking of an infected patient or by aerosols (particles under 100 μm in diameter) formed via surgical and dental procedures 11,12. The exposure and, hence, risk of transmission are increased if the infected person is present within a 1-m length of the susceptible host 13.

### Fecal-oral transmission

Many reports have stated the presence of gastrointestinal symptoms throughout the course of the disease as well as the existence of viral RNA in the fecal specimen 14. In the gastrointestinal tract, enterocytes highly express angiotensin-converting enzyme 2 (ACE2) and transmembrane protease serine 2 (TMPRSS-2) receptors, which are targets of SARS-CoV-2 cell entry 15. The gastrointestinal symptoms (e.g., nausea, vomiting, diarrhea, anorexia, and abdominal discomfort) during the course of COVID-19 might be due to infection of the enterocytes by SARS-CoV-2 16. The phenomenon of the gut lung axis is another speculated cause, where these gastrointestinal changes in COVID-19 might be a secondary effect of the major pulmonary changes, or SARS-CoV-2 might infect enterocytes, which might lead to gut dysbiosis and might cause increased damage to the lungs 17.

### Vertical transmission

Research has identified that pregnant women are more susceptible to viral infections due to physiological adaptations (immunologic and anatomic alterations), which increase the risk of developing severe illness 18. Currently, the pathophysiology underlying this increased morbidity and its potential impact on the developing fetus is of particular concern when mothers are infected with SARS-CoV-2. As recently reported, the two known SARS-CoV-2 receptors ACE2 and TMPRSS-2 are widely spread in specific cell types of the maternal-fetal interface and fetal organs 19,20. Therefore, the impact of the virus on the placenta and the potential for the vertical transmission of SARS-CoV-2, although rare, is possible and apparently related to a high maternal and fetal inflammatory state. Although further studies are needed and no firm conclusions can be drawn due to the low number of analyzed cases, this should be taken into consideration in the management of pregnant women with COVID-19 21, 22.

### Ocular transmission

When exposed to a contaminated environment, the ocular surface is another probable location of viral infection. In anatomical theory, Belser et al. described respiratory disease spread through the eye by means of the nasolacrimal system 23. As a result, numerous respiratory virus-infected aerosols and hands contact the eye surface, and viruses can later use the eye as a site of virus replication and enter the respiratory system through the nasolacrimal system. As a mediator for virus entry into host cells, ACE2 and CD147 (BSG) are also expressed in the retina, thus, it is possible that SARS-CoV-2 can harm the retina 24, 25. With the ongoing outbreak, mounting evidence suggests a relationship between SARS-CoV-2 infection and ocular involvement 26.

## Specimen type of nucleic acid testing

The detection of SARS-CoV-2 using nucleic acids provides direct evidence for COVID-19 diagnosis. Specimen choice from different groups of people, including mild to severe COVID-19 patients, suspected cases, asymptomatic carriers, close contacts and mass screening, was examined to improve the accuracy of detection (Table 1).

## Anal swab for SARS-CoV-2 screening

Patients infected with SARS-CoV-2 may harbor the virus in the intestine at the early or late stage of COVID-19 and present with SARS-CoV-2 RNA-positive stool samples, and the respiratory RNA test results may not be consistent with those from stool samples. Notably, the team of Shi et al. from Wuhan Institute of Virology, Chinese Academy of Sciences first proposed the necessity of an “anal swab” for nucleic acid testing in Feb 2020 27. They investigated on patients in a local hospital who were infected with SARS-CoV-2 and found the presence of SARS-CoV-2 in anal swabs and 75% (6/8) anal swabs were positive compared to 4/8 (50%) oral swabs that were positive in a later stage of infection, suggesting virus shedding and thereby transmission through the oral-fecal route. Therefore, the strategy for detecting viral RNA in oropharyngeal swabs is not perfect. Simultaneously, Xiao et al. also observed that the test result for viral RNA remained 23% (17/73) positive in feces, even after test results for viral RNA in the respiratory tract converted to negative, indicating that the viral gastrointestinal infection and potential fecal-oral transmission can last even after viral clearance in the respiratory tract. Therefore, they strongly recommend that RT-PCR testing for SARS-CoV-2 from feces should be performed routinely in patients with SARS-CoV-2 28. According to a retrospective cohort study in Zhejiang, China, viral RNA was detected in the stool of 59% (55/93) of patients. The median duration of viral RNA in stool was 22 days. The duration of SARS-CoV-2 infection is significantly longer in stool samples than in respiratory and serum samples, highlighting the need to strengthen the management of stool samples in the prevention and control of the epidemic 29. Notably, eight of ten pediatric SARS-CoV-2 infection cases persistently tested positive on rectal swabs even after nasopharyngeal testing turned negative, especially in asymptomatic children 30. Moreover, prolonged fecal elimination, lasting dozens of days after negative results from RT-PCR assays on respiratory swabs, has been reported in all 4 children with SARS-CoV-2 infection 31. These observations raise the question regarding the possibility of oral-fecal transmission and the possible role of children in spreading the infection, particularly when they appear asymptomatic or with gastrointestinal symptoms but with no respiratory involvement 32. In the current case series, up to 18% of patients with COVID-19 had gastrointestinal symptoms. Viral RNA was detected in fecal samples from 40.5% (1946/4805) and 48.1% (2041/4243) of patients with COVID-19, even in stool collected after respiratory samples had negative test results, suggesting that the digestive tract might be another site for viral replication and activity 33,34. McDermott et al. suggested the importance of early recognition of gastrointestinal tract signs, sometimes the only presenting symptoms, by clinicians and infection control professionals. Such recognition is critical to avoid delayed diagnosis and unintended transmission events in hospital settings 35. Notably, Lin et al. suggested that detectable SARS-CoV-2 in the digestive tract was a potential warning indicator of severe disease. Screening the virus in the digestive tract, close monitoring, and early intervention in patients with the detectable virus are needed 36.

Very recently, SARS-CoV-2 genetic variation and the arrival of the winter season have enhanced infectivity and have put much pressure on the prevention and control of the epidemic. Small, localized outbreaks and a number of cases imported from other countries have occurred in China. Anal swabs are now regarded as a complementary sample to nasopharyngeal swabs. As a proof of this, on Jan 20, 2021, the Conference on Beijing COVID-19 Epidemic Prevention and Control announced that anal swabs were listed as one of the collection methods for key population screening in the Daxing district of Beijing in an effort to contain the latest COVID-19 resurgence. This news came as Daxing district reported locally transmitted cases. According to Spanish media reports, Spain's Galicia region has started anal swab testing for SARS-CoV-2. The anal swab method can increase the detection rate of infected people as traces of the virus linger longer in the anus than in the respiratory tract. Respiratory swabs may make these patients easy to ignore 37. As a promising sampling complement, anal swabs are equivalent to providing “double insurance” for nucleic acid testing to prevent missed detection.

## Conclusions

The role of anal swab sampling during the COVID-19 pandemic has received increasing attention from researchers for several reasons. First, based on the analysis of a relatively large number of samples collected from severe COVID-19 patients, a high viral RNA positivity rate in anal swabs, a high viral load in anal swabs, and early positive detection in anal swabs can predispose COVID-19 patients to adverse outcomes. The presence of viral replication in extrapulmonary sites predisposes patients to adverse outcomes and should thus be monitored carefully 38. Second, using anal swab sampling, the risk of viral transmission for health care workers who perform the procedure is dramatically reduced in the field (point-of-need). Third, anal swab specimens could be an option for young children, for whom it is difficult to collect a reliable respiratory sample. Finally, the study showed that the detection rate of nasopharyngeal swabs and oropharyngeal swabs was higher before washing in the morning and was relatively low in the afternoon; anal swabs collected might prevent missed detection 39. To achieve scientific and precise prevention and control, an anal swab nucleic acid test is necessary, which can increase the positive diagnostic rate of COVID-19 infection patients at the late infection stage, reduce the probability that patients turn positive again after discharge and screen key populations to improve the effectiveness of public health interventions to limit future pandemics. This study was not systematically conducted, and the data presented were made available according to the authors' preference and may be subject to bias or missing data; thus, further research is needed.

## Figures and Tables

**Table 1:** Specimen type of nucleic acid testing.

Infection pathway	Type	Specimen collecting
Upper respiratory tract	Np swab	Tilt the patient's head slightly backward. The distance between the tip of the nose and the ear lobe is precisely measured with a swab and marked with a finger. Insert the swab to the measured distance. Leave the swab in the nose for 15-30s, gently rotating 3-5 times then immediately place it in the sample collection tube filled with 2 ml lysate or a cell preservation solution containing the RNase inhibitor.
	OP swab	It is recommended that a sterile flock swab be used for sampling by wiping the back wall of the pharynx with moderate force. During the process, touching the tongue should be avoided. The swab should be placed into the same collection tube as the Nasopharyngeal swab.
	Saliva	The oral cavity was cleaned by normal saline, then collected about 1.5 ml of midstream salivary fluid in screw-capped specimen collection tubes.
Lower respiratory tract	Deep cough sputum	Deep cough sputum should be collected in a disposable sterile screw-cap sampling cup containing 2 ml of proteinase K, closing the container upon collection. The test should be conducted within 30 min if possible. If the specimen needs to be transported over a long distance, proteinase K should not be added in advance.
	BALF	In the case of severe patients or patients with rapidly progressing pneumonia, the clinician should aseptically collect ≥5 ml BALF into a 50-ml sterile container.
Digestive tract	Anal swab	A sterile cotton swab is inserted into the anus 3-5 cm, gently rotating swab then immediately collected in screw-capped specimen collection tubes (RNase inhibitors added if possible).

*Abbreviations:* NP: nasopharyngeal; OP: oropharyngeal; BALF: bronchoalveolar lavage fluid.
